# Diplomas and Certificates Granted at Edinburgh and London, 1835-6

**Published:** 1837-01

**Authors:** 


### DIPLOMAS GRANTED BY THE COLLEGE OF SURGEONS OF EDINBURGH, 1835-6.

The number of gentlemen who have obtained the diploma of the Royal College of Surgeons of Edinburgh between the 31st of August, 1835, and 1st of September, 1836, is 155: viz. 103 from Scotland; 16 from England; 22 from Ireland; and 14 from the colonies.

---

### DIPLOMAS GRANTED BY THE COLLEGE OF SURGEONS OF LONDON, 1835-6.

The number of gentlemen who have passed their examination at the College, and obtained diplomas, from September 1835 to September 1836, is 463.

---

### CERTIFICATES GRANTED BY THE APOTHECARIES' COMPANY, 1835-6.

The number of gentlemen who have passed their examinations at Apothecaries' Hall, and obtained certificates for practice, from October 3, 1835, to September 29, 1836, is 450: of this number eighty only obtained the diploma from the College of Surgeons.

